# High prevalence of chitotriosidase deficiency in Peruvian Amerindians exposed to chitin-bearing food and enteroparasites

**DOI:** 10.1016/j.carbpol.2014.07.011

**Published:** 2014-11-26

**Authors:** N. Manno, S. Sherratt, F. Boaretto, F. Mejìa Coico, C. Espinoza Camus, C. Jara Campos, S. Musumeci, A. Battisti, R.J. Quinnell, J. Mostacero León, G. Vazza, M.L. Mostacciuolo, M.G. Paoletti, F.H. Falcone

**Affiliations:** a Department of Biology, Università degli Studi di Padova, Padova, Italy; b Facultad de Ciencias Biologicas, Universidad Nacional de Trujillo, Trujillo, Peru; c Division of Molecular and Cellular Science, School of Pharmacy, University of Nottingham, Nottingham, United Kingdom; d Facultad de Ciencias Sociales, Universidad Nacional de Trujillo, Trujillo, Peru; e Dpt. of Chemical Sciences, Università di Catania and Institute of Biomolecular Chemistry, CNR, Catania, Italy; f Department DAFNAE, Università degli Studi di Padova, Legnaro, Padova, Italy; g School of Biology, University of Leeds, Leeds, United Kingdom

**Keywords:** Human chitotriosidase, Amazonian Amerindians, Exposure to chitin, Chitin-bearing parasites, 24-bp duplication/H-allele, Single nucleotide polymorphisms

## Abstract

The human genome encodes a gene for an enzymatically active chitinase (*CHIT1*) located in a single copy on Chromosome 1, which is highly expressed by activated macrophages and in other cells of the innate immune response. Several dysfunctional mutations are known in *CHIT1*, including a 24-bp duplication in Exon 10 causing catalytic deficiency. This duplication is a common variant conserved in many human populations, except in West and South Africans. Thus it has been proposed that human migration out of Africa and the consequent reduction of exposure to chitin from environmental factors may have enabled the conservation of dysfunctional mutations in human chitinases. Our data obtained from 85 indigenous Amerindians from Peru, representative of populations characterized by high prevalence of chitin-bearing enteroparasites and intense entomophagy, reveal a very high frequency of the 24-bp duplication (47.06%), and of other single nucleotide polymorphisms which are known to partially affect enzymatic activity (G102S: 42.7% and A442G/V: 25.5%). Our finding is in line with a founder effect, but appears to confute our previous hypothesis of a protective role against parasite infection and sustains the discussion on the redundancy of chitinolytic function.

## Highlights

• Catalytic deficiency of chitotriosidase has a very high frequency in Amerindians highly exposed to chitin from enteroparasites and diet.
• Mutation frequencies are similar to those found in East Asian populations, and is probably conserved for a founder effect.
• Such condition precludes the use of CHIT1 as a disease biomarker in South American populations with strong ethnic ancestry.

## 1 Introduction

Due to its unique biomechanical properties, chitin is one of the most abundant biopolymers in the biosphere (Muzzarelli et al., 2012, Musumeci & Paoletti, 2009) constituting structures with defensive (fungal cell wall, nematode egg shell), predatory (hooks) or nutritional (pharynx, mollusc radula) functions in many eukaryotic organisms, such as protozoans, insects and nematodes (Muzzarelli, 2011, Zakrzewski et al., 2014). While humans do not have the ability to synthesize chitin, they are known to produce chitinolytic enzymes. Chitotriosidase (CHIT1, or macrophage chitinase), together with acidic mammalian chitinase (AMCase) is one of the two known human enzymes able to cleave chitin. It is highly expressed by activated macrophages (Van Eijk et al., 2005) and is pre-formed in granules of neutrophils (Boussac & Garin, 2000). At the tissue level, CHIT1 is expressed in the human lung (Seibold et al., 2008), human lachrymal glands (Hall, Morroll, Tighe, Götz, & Falcone, 2008) and in both bone marrow and spleen of mice (Boot et al., 2005).

The expression in key innate immune cells and tissues at the host/environment interface is strongly suggestive of an involvement in innate immunity, for example against chitin-bearing pathogens such as fungi. Indeed such a protective function is well known to be an important element of immunity in plants, where chitinases are found amongst the so-called pathogenesis-related proteins (Kasprzewska, 2003). There is also increasing evidence for a role in innate immunity in mammals, particularly against fungal pathogens. Indeed, CHIT1 inhibits pathogenic chitin-producing fungi including *Candida albicans* (Vandevenne et al., 2011), *Aspergillus niger* and *Cryptococcus neoformans* (Gordon-Thomson et al., 2009), though it was concluded that it is less effective than lysozyme in restricting the growth of fungal pathogens (Vandevenne et al., 2011). Based on the chitinolytic activity of lysozyme (Marquis, Montplaisir, Garzon, Strykowski, & Auger, 1982), Hall and co-authors postulated a synergistic action between CHIT1 and lysozyme, but could not find any evidence for this in antibacterial immunity *in vitro* (Hall et al., 2008). More recently, a synergistic action of both mammalian chitinases in antifungal immunity has been demonstrated in a rat model of aspergillosis (although this required disruption of the cell wall with caspofungin) (Verwer et al., 2013), a situation reminiscent of the concerted action of chitinases and β-1,3-glucanases in antifungal plant immunity (Jongedijk et al., 1995).

Natural mutations which disrupt functionality can give insights into the roles of human genes, as can the use of gene-disrupted animal models. Several dysfunctional mutations in *CHIT1* have been found to be prevalent in human populations, without the association of any evident phenotype, suggesting that CHIT1 function is partially redundant (Boot et al., 1998). Indeed, a 24-bp duplication in Exon 10 of the chitotriosidase gene, causing the loss of the catalytic domain, is highly conserved in many human populations, but has not been found in primates, suggesting that it is a post-speciation event (Gianfrancesco & Musumeci, 2004). Specifically, this variant, also named H-allele, is almost absent in some West African (Burkina Faso: 0.2%) (Malaguarnera et al., 2003) and South African (South Africa: 0%) (Arndt, Hobbs, Sinclaire, & Lane, 2013) populations and showed the highest frequencies in Asiatic populations, suggesting it may have arisen after human migration out of Africa (Piras et al., 2007a, Piras et al., 2007b).

Previous studies have hypothesized that the difference in duplication frequencies found between African populations in Benin, Burkina Faso and South Africa (Arndt et al., 2013, Malaguarnera et al., 2003) (98–100% homozygous wild-type) and those found in European populations, e.g. in Corsica and Sardinia (Piras et al., 2007a, Piras et al., 2007b), Spain (Irún, Alfonso, Aznarez, Giraldo, & Pocovi, 2013), Portugal (Rodrigues, Sá Miranda, & Amaral, 2004) and the Netherlands (Boot et al., 1998) (<77% homozygous wild-type) may be due to the greater prevalence of parasitic infections in African populations, suggesting that chitotriosidase may possess an anti-parasitic function which has led to the maintenance of the wild-type allele in endemic areas. Overall, the frequency of the H-allele appears to vary significantly between populations (Arndt et al., 2013, Boot et al., 1998, Hise et al., 2003, Malaguarnera et al., 2003, Woo et al., 2014) and this variance in the frequency of functional chitotriosidase suggests that different populations vary in their need for the active protein. However, several studies could not find any correlation between rates of parasitic infection and duplication frequency in non-African areas endemic for parasitic infections (Hall et al., 2007, Hise et al., 2003).

We were therefore interested in studying *CHIT1* genotype frequencies in a South American indigenous population with very low genetic admixture and very high exposure to chitin, through parasites and food, reflecting an ancestral lifestyle.

## 2 Materials and methods

### 2.1 Sample characterization

#### 2.1.1 Ethical statement

Biological saliva specimens were taken safely and non-invasively, in full compliance with protocols approved by the Ethics Committee of the Università di Padova (2008). Informed consent was obtained from volunteers, or from their parents for underage volunteers. Project aims were presented to, and informed consent approved by, *Awajún* and *Ashaninka* indigenous organizations: OCCAAM (Central Organization of Awajún Communities of Alto Marañon) and ANAP (Pichis River Ashaninka Nationalities Association), respectively.

#### 2.1.2 Peruvian Amerindians

In the Peruvian Andes and Amazons a high ethnic diversity is still preserved. Amerindians live in small communities of fifty up to several hundred people, and still maintain their original languages and bio-cultural adaptation to specific environmental conditions. Until the 1970s, most Amazonian communities of Peru were geographically isolated as they were cut off from the main routes of transportation, showing the highest prevalence of parasites and the lowest levels of water sanitation and national health-care of the country (Instituto Nacional de Salud, 2000, MINSA/OGE, 2002, MINSA/OGE, 2003).

**Fig. 1:**
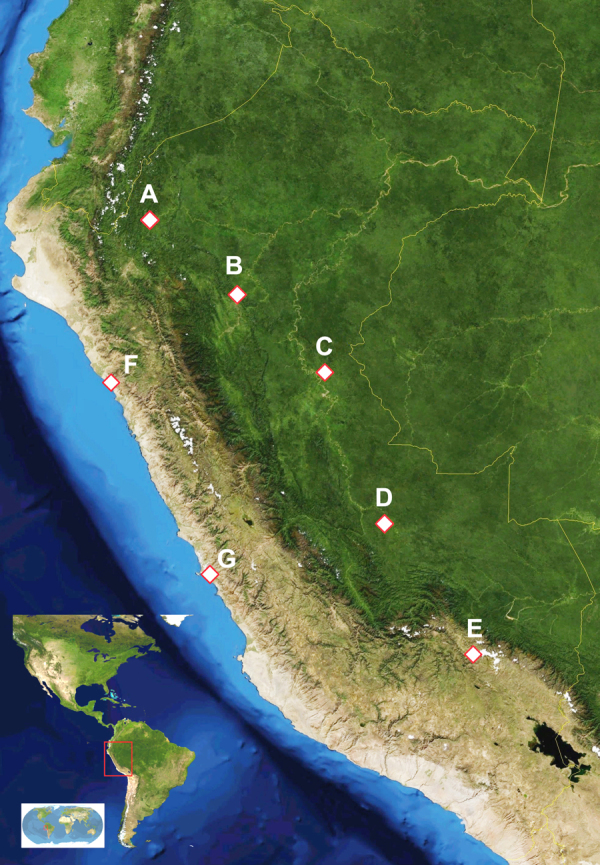
Satellite map of Peru indicating sampling locations: *Awajùn* (A); *Quechua-Lamas* (B); *Shipibo* (C); *Ashaninka* (D); *Quechua-Cusco* (E); non-indigenous controls from Trujillo (F), and Lima (G). *Shipibo* people are residents in Cantagallo slum, Lima, from 2000–2001 onwards, people >10 years old were born in original NCs in Ucayali Region (C).

Ethnic Amerindians involved in this study belong to five ethnic groups (Fig. 1):

- ***Awajún*** of *Rio Marañon*; Native Communities (NCs): Tzuntsuntsa, Yamayacat and Putuim (Amazonas Region); linguistic family: *Jìbaro*;
- ***Ashaninka*** of *Rio Pichis* and *Perené*; NCs: San Juan (Pasco Region), Puerto Ocopa and Pangà (Junín Region); linguistic family: *Arawaks*;
- ***Shipibo-Conibo*** of *Rio Ucayali* (Ucayali Region), recently (2000–2002 A.D.) migrated to Lima; linguistic family: *Pano*;
- ***Quechua-Lamas***; NC: Lamas, with an Andean ancestry, now living in the high Amazon (San Martìn Region); linguistic family: *Quechua*;
- ***Quechua-Cusco***; NC: Huilloq (Cuzco Region), living in Andean highlands (altitude: 3000–4000 m above sea level); linguistic family: *Quechua*.

The populations are reciprocally isolated by both cultural (linguistic) and geographical barriers (see reciprocal distances in Table 1; mean: 1356 km), but, because of the small sample, the five Amerindian populations were considered as subpopulations and genetic data were finally clustered and discussed together, as ‘Amerindian’ population.

**Table 1:** Geographic distance between native communities considered in this study.

	Quechua-Cusco	Ashaninka	Shipibo	Quechua-Lamas
Awajún	2419	1835	1247	**610**
Quechua-Lamas	2289	1702	**1117**	
Shipibo	1172	**585**		
Ashaninka	**587**		Mean: 1.356 km	

Distances are calculated by GoogleMaps, lowest reciprocal distances are in bold.

#### 2.1.3 Assessment of exposure to chitin

As indicators of environmental exposure to chitin we used enteroparasitic prevalence and chitinous food consumption. The ingestion of chitin containing foods, such as crustaceans, insects and fungi, was assessed in the Amerindian and urban sample by standardized interviews and qualitative observations of ethnobiological habits.

Between December 2010 and March 2013, in *Awajún*, *Ashaninka*, *Shipibo* and *Quechua-Cusco*, the presence of intestinal parasites was assessed in a total sample population of 90 (from 14 to 34 volunteers for each NC: *Awajún n* = 34; *Ashaninka n* = 21; *Shipibo n* = 21; *Quechua-Cusco n* = 14) (5–12% of total villagers). Faecal samples were conserved in a 10% formol solution and analysed in the Helminthology Laboratory of Universidad Nacionàl de Trujillo, using standard protocols for direct copro-parasitological analysis (Instituto Nacional de Salud, 2003). Faecal samples were treated either by ether-sedimentation (Ritchie, 1948) or sugar-flotation (Sheather, 1923) preparation methods, and were examined with a binocular microscope (60–100× magnification), in duplicate, for detection and identification of eggs and cysts. For urbanized controls, epidemiological information was collected at local health institutions.

### 2.2 Genetic study

#### 2.2.1 Genetic sampling

Genetic sampling was carried out with 85 ethnic Amerindian subjects and 50 urban controls. Saliva samples were collected with Oragene™ DNA Self-collection Kit (DNA Genotek, Canada) and DNA extracted as directed by the manufacturer. This device guarantees sample conservation at room temperature in hot and wet climatic conditions. The control population of 50 subjects was sampled in urban areas of Peru: Trujillo (*n* = 42) and Lima (*n* = 4) on the coast, and Tarapoto city (*n* = 4), in the Amazon. Most non-ethnic controls declared an Andean ancestry. Kinship relation between the volunteers was assessed by personal interviews and general pedigree and family trees were reconstructed, in the whole sample.

Unfortunately, volunteers involved in genetic and parasitological study rarely coincided in our study, because the two samplings were realized during different expeditions to the various remote communities; moreover, because of explicit requests by village leaders, parasitological analysis had to involve school-age children with suspected malnutrition while, contrarily, genetic sampling interested almost exclusively adult or elderly individuals.

#### 2.2.2 Screening of the 24-bp duplication

For genetic screening of DNA samples for the 24 base pair duplication PCR was conducted in 15 μl reactions consisting of 0.25 U of FastStartTaq (Roche), 2 mM MgCl_2_ buffer, 0,2 μl *Forward Primer* 5′-CCTGTCCAGAAGAGGTAGCC-3′, 0,2 μl of *reverse primer* 5′-CCTCCAAATTCCACCACTG-3′, 200 μM dNTPs, 1 μl of genomic DNA, and 9 μl of nuclease-free water. Primers were used at 250 nM final concentration. The Touchdown PCR program used was as follows: initial denaturation 94 °C for 4 min, followed by 10 cycles [94 °C for 40 s (denaturation) + 70 − 1 °C for 40 s (annealing) + 72 °C for 40 s (elongation)], 33 cycles [94 °C for 40 s, 60 °C for 40 s, 72 °C for 40 s], and a final extension at 72 °C for 7 min. Detection of 24-bp duplication on the PCR products was performed by DNA sequencing and/or by fragment separation by agarose gel electrophoresis in 4% standard molecular biology grade agarose gels (Sigma-Aldrich, UK) or on 3.5% MetaPhor™ Agarose (Lonza).

#### 2.2.3 Polymorphism analysis for G102S and A442G/V SNP

G102S (rs2297950) and A442G/V (rs1065761) SNPs were analysed by PCR-RFLP using primers previously described by Bierbaum, Superti-Furga, and Heinzmann, (2006). HpaII or HinP1L restriction sites (for G102S and A442G/V SNPs, respectively) were inserted via site directed mutagenesis completed during PCR of 30 μl reactions consisting of 15 μl REDTaq^®^ ReadyMix™ PCR Reaction Mix (Sigma-Aldrich), 10.5 μl nuclease-free water, 1.5 μl CHIT1 Exon 4 forward primer 5′-CCATCGGAGGCTGGAATTCC-3′, 1.5 μl CHIT1 Exon 4 reverse primer 5′-TCTGGCAAGACTGGATCTGA-3′ for G102S, or CHIT1 Exon 11 Forward Primer 5′-TGTGGGTTTGGGATCTCTTC-3′, 1.5 μl CHIT1 Exon 11 reverse primer 5′-TTTGCTGGAACAGCCGCCGC-3′ for A442G/V, and 1.5 μl of genomic DNA sample, utilizing the following cycling conditions. Initial denaturation 94 °C for 5 min, followed by 35 cycles of denaturation at 94 °C for 30 s, annealing at 56 °C (G102S) or 52 °C (A442G/V) for 45 s, and elongation at 72 °C for 1 min 30 s, followed by a final extension at 72 °C for 5 min. All primers were used at 250 nM final concentration.

**Fig. 2:**
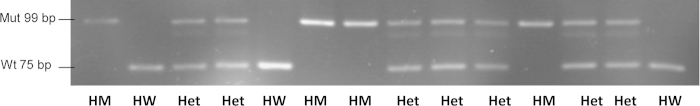
Example of high resolution gel electrophoresis screening for CHIT1 24 base pair duplication following PCR. Genomic DNA was amplified using primers described by (Boot et al., 1998) PCR products were separated on 3.5% high resolving agarose. Higher 99 bp bands indicate allele containing the 24 bp duplication, lower 75 bp bands indicate the wildtype allele (no duplication). A single higher band indicates homozygous mutant (HM), a single lower band indicates homozygous wild type (HW) and the presence of both bands indicates heterozygosity (Het).

**Fig. 3:**
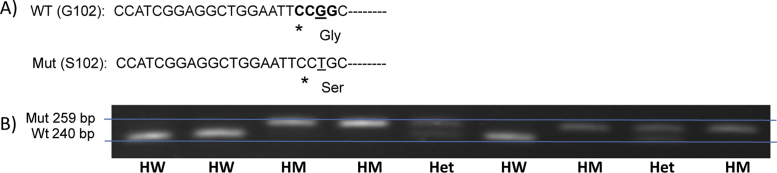
(A) Illustration of the principle underlying PCR-RFLP typing of the G102S SNP (rs2297950). Primers described by Bierbaum et al. (2006) result in amplification of a 259 bp gene fragment from genomic DNA located in Exon 4 and the following intron of the CHIT1 gene. The forward primer introduces a mutation (T → C, denoted by an asterisk) located just 2 nucleotides upstream of the position of the SNP, introducing an Hpa II restriction site (C′CGG, bold characters) for the wildtype G102 SNP (resulting in a 240 bp and a 19 bp fragment after restriction) but not the mutant S102 SNP (uncleaved 259 bp fragment). (B) Example of gel electrophoresis screening for CHIT1 G102S SNP following PCR and digestion with Hpa II. Higher bands indicate undigested allele containing S102 SNP, lower bands indicate large fragment of digested wild G102 allele. Single high band indicates homozygous mutant (HM), single lower band indicates homozygous wild type (HW) and presence of both bands indicates heterozygosity (Het).

**Fig. 4:**
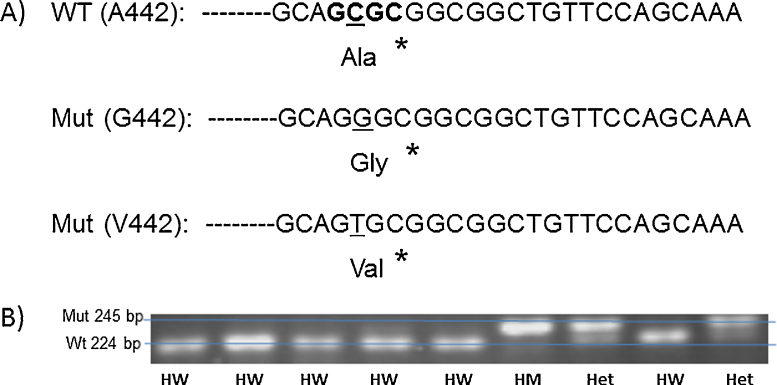
(A) Illustration of the principle underlying PCR-RFLP typing of the A442G/V SNP (rs1065761). Primers described by Bierbaum et al. (2006) result in amplification of a 245 bp gene fragment from genomic DNA located in Exon 12 of the CHIT1 gene. The reverse primer introduces a mutation (G → C, denoted by an asterisk) located just two nucleotides downstream of the position of the SNP, introducing an Hinp1L restriction site (G′CGC, bold characters) for the wildtype A442 allele (resulting in a 224 bp and a 21 bp fragment after restriction), but not the A442G/V SNP (uncleaved 245 bp fragment). (B) Example of gel electrophoresis screening for CHIT1 A442G/V SNPs following PCR and digestion with Hinp1L. Higher bands indicate undigested mutant allele A442G or A442V, lower bands indicate A442 wildtype. Single high band indicates homozygous mutant (HM), single lower band indicates homozygous wildtype (HW) and presence of both bands indicates heterozygosity (Het).

Amplicons were then purified with a Wizard^®^ SV Gel and PCR Clean-Up System (Promega) according to the manufacturer's protocol. Resultant elutes were then digested with 1 μl HpaII (for G102S) or HinP1I (A442G/V) restriction enzyme (both from New England Biolabs) according to the manufacturer's instructions. Resultant digestions were then separated via gel electrophoresis run on 4% agarose (Sigma-Aldrich) or 3.5% MetaPhor™ Agarose (Lonza). The distinction between wild-type, heterozygous and homozygous mutant genotypes was made based on the size and number of resultant fragments. Details of fragment sizes and examples are shown in Fig. 2, Fig. 3, Fig. 4.

### 2.3 Data analysis

Genotypic frequencies were compared with frequencies expected by Hardy-Weinberg equilibrium (HWE). Based on general pedigree data, inbreeding coefficients and kinship were calculated by KinInbcoef program (Bourgain, 2005), and allelic frequency values were corrected by kinship degree.

## 3 Results

### 3.1 Exposure to chitin: Diet and enteroparasites

Ethnobiological information confirmed traditional dietary habits including daily consumption of insects, crustaceans and mushrooms, and a very rare presence of pets, in the Amerindian sample, especially in *Awajún*, *Ashaninka*, *Quechua-Lamas* and *Shipibo* (before migrating to urban Lima). In contrast, *Quechua-Cusco* people reported only rarely consuming insects, crustaceans and mushrooms. Similarly, urban controls declared to eat, weekly, crustaceans, and, very rarely, mushrooms.

The three Amazonian communities (*Awajún*, *Ashaninka* and *Shipibo*) suffer a very high prevalence of enteroparasites (Table 2): 19–43% for nematodes, 35–62% for protozoans and 3–29% for *Hymenolepis nana* (the only cestode found in our sample). Also biparasitism (21.1%) and tetraparasitism (6.7%) rate was high in Amazonians. In *Quechua-Cusco* community we found predominantly cases of *Entamoeba histolytica* and *Ascaris lumbricoides*. The main chitin-bearing parasites (based on published literature (Arroyo-Begovich et al., 1980, [Bibr bib0060][Bibr bib0130], Lanuza et al., 1996, Wimmer et al., 1998, Zhang et al., 2005) present in the sample population were the protozoans *Giardia*, *Entamoeba histolytica*, and the macro-parasites *Ancylostoma/necator*, *Ascaris*, *Enterobius vermicularis* and *H. nana*.

**Table 2:** Prevalence of enteroparasites in Peruvian Amerindians.

	Ashaninka (*n* = 21)	Awajùn (*n* = 34)	Shipibo (*n* = 21)	Quechua-Cusco (*n* = 14)
Entamoeba coli*	0.09	0.18	0.24	0.64
Entamoeba histolytica*	0.14	0.03	0.19	0
Blastocystis hominis*	0.24	0.20	0	0
Giardia lamblia*	0.24	0.12	0.33	0.07
Ascaris lumbricoides*	0.28	0.26	0.14	0.14
Enterobius vermicularis*	0.14	0	0	0
Strongyloides stercoralis*	0	0	0.05	0
Trichuris trichiura*	0	0	0	0
Ancylostoma/necator*	0.09	0.09	0	0
*Taenia* spp.*	0	0	0	0
Hymenolepis nana*	0.09	0.03	0.28	0
*Paragonimus* spp.	0	0	0	0
*Schistosoma mansoni*	0	0	0	0
*Fasciola hepatica*	0	0	0	0
By phylum				
Protozoa*	0.62	0.35	0.57	0.71
Nematoda*	0.43	0.32	0.19	0.14
Cestoda*	0.09	0.03	0.28	0
Trematoda	0	0	0	0

Parasite prevalence is calculated as frequency of infected individuals for each community sampled. Prevalence is given by species (or by phyla) in the four communities studied.

* Parasites thought to be chitin-bearing are marked with asterisk.

In contrast, lower parasite prevalence of mainly protozoan parasites characterizes the control population of urbanized Peruvians.

### 3.2 Chitotriosidase deficiency

The 24-bp duplication causing catalytic deficiency was highly prevalent (Table 3), with a frequency of 47.06% (Kinship correction: 45.99%) in the whole indigenous population, and 27.55% (Kinship correction: 28.88%) in non-indigenous controls. Genotypic frequencies were consistent with the Hardy-Weinberg Equilibrium (HWE), within each indigenous population, in total Amerindians, and controls.

**Table 3:** Frequencies of wild type and the 24-bp duplication in 135 Peruvians.

Population	*n*	Genotype frequency			Allele frequency		*χ*^2^	HWE *p* value
		*Wt/Wt* (%)	*Wt/Mut* (%)	*Mut/Mut* (%)	*Wt* (%)	*Mut* (%)		
Indigenous Amerindians	85	28	49	22	53	47	0.006	0.939
non-indigenous	50	58	30	12	73	27	2.855	0.091
Amazonian indigenous	60	26	47	26	50	50	0.267	0.606
Quechua indigenous	25	32	56	12	60	40	0.694	0.405

Results are shown for indigenous samples (*n* = 85) vs. non-indigenous controls (*n* = 50), as well as for Amazonian (*n* = 60) vs. Quechua (*n* = 25) indigenous samples. Wt indicates wildtype (functional) CHIT1 genotype, Mut the mutant (non-functional 24 bp duplication/H-allele) CHIT1 genotype. Wt/Wt and Mut/Mut are homozygous, whereas Wt/Mut are heterozygous carriers of this gene.

Next, we screened a subset (unselected) of 55 indigenous Peruvians (33 Amazonians and 22 *Quechua*) and 28 Peruvian non-indigenous controls for previously described non- synonymous SNPs in the *CHIT1* gene, G102S and A442G (Bierbaum et al., 2006) as well as A442V (Lee, Waalen, Crain, Smargon, & Beutler, 2007). The method used does not discriminate A442G from A442V; hence results are given collectively as A442G/V for the latter SNP (Table 4).

**Table 4:** Allelic frequencies for G102S SNP and A442G/V SNPs in 83 Peruvians.

SNP	Genotype frequency			Allele Frequency		*χ*^2^	HWE *p* value
	*Wt/Wt*	*Wt/Mut*	*Mut/Mut*	*Wt*	*Mut*		
Indigenous samples (*n* = 55)							
*G102S*	34.55%	45.45%	20.00%	0.573	0.427	0.005	>0.995
*A442G/V*	56.36%	36.36%	7.27%	0.745	0.255	1.626	0.975
Non-indigenous controls (*n* = 28)							
*G102S*	25.00%	53.57%	21.43%	0.518	0.482	0.005	0.995
*A442G/V*	46.43%	50.00%	3.57%	0.714	0.286	2.488	0.975
Amazonian indigenous (*n* = 33)							
*G102S*	45.45%	36.36%	18.18%	0.636	0.364	0.046	0.995
*A442G/V*	48.48%	39.39%	12.12%	0.682	0.318	0.008	>0.995
Quechua indigenous (*n* = 22)							
*G102S*	18.18%	59.09%	22.72%	0.477	0.523	0.034	0.995
*A442G/V*	68.18%	31.81%	0.00%	0.841	0.159	0.036	0.995

Results are shown for indigenous samples (*n* = 55) vs. non-indigenous controls (*n* = 28) as well as for Amazonian (*n* = 33) vs. Quechua (*n* = 22) indigenous samples. Wt indicates wildtype (G102 or A442) CHIT1 genotype, Mut the SNP (102S or 442G/V, as indicated in the left column) CHIT1 genotype. Wt/Wt and Mut/Mut are heterozygous, whereas Wt/Mut are homozygous carriers of this gene.

Similarly to the results obtained for the H-allele, the genotype screening for the G102S SNP showed a high frequency for the mutation, while the frequency of A442G/V was somewhat lower. There was no significant departure from HWE in any population.

## 4 Discussion

Both ethnobiological and parasitological studies gave evidence of a conserved traditional lifestyle, strongly influenced by environmental factors and, specifically, by chitin-containing parasites and traditional local food including chitin-bearing invertebrates and mushrooms. The absence of *Taenia* and *Fasciola* is most likely related to the very low presence of livestock in indigenous Amazonian areas, where ‘original’ ethno-ecological features regarding nutrition and lifestyle are still predominant. Overall, the absence of such zoonoses, together with a high prevalence of soil-transmitted helminths (nematodes) can be considered an ancestral parasitological condition, as confirmed by pre-Columbian fossil remains (Araujo, Reinhard, Ferreira, & Gardner, 2008; Sianto et al., 2009). Furthermore, the very low prevalence of filarial nematodes in the Amazon is a rare feature for worldwide tropical areas, confirming a broadly ‘better’ parasitological condition/reduced parasitological burden, in original Amerindians.

Although no correlation analysis between *CHIT1* genotype and parasite infection is possible with present data, our key finding is that the prevalence of chitotriosidase deficiency in the indigenous sample is very high, with up to 26% of individuals having no functional chitotriosidase. Thus, we can conclude that chitin-containing enteroparasites are not exerting any significant selective pressure for functional chitotriosidase in our sample. The prevalence of chitotriosidase deficiency is lower in non-indigenous controls, which is likely to result from genetic admixture with Europeans and Africans in recent times.

The genotype screening for the G102S SNP similarly shows a high frequency for the mutation (allelic freq. = 36–52%), though in this case allele frequencies were similar in indigenous and non-indigenous samples, but higher compared to other human populations (allelic freq. = 27–37%) (data available from the literature (Lee et al., 2007), and from ENSEMBL genomic database: http://www.ensembl.org/index.html; ref seq.: rs2297950). For the A442G/V SNPs, the mutant allele frequency (16–32%) is lower compared to the G102S SNP, but still higher compared to the frequencies (allelic freq. = 7–17%) reported for other populations (ENSEMBL, ref. seq.: rs1065761). Previous studies have indicated that the effect of the G102S SNP on enzymatic activity varies from undetectable to significant, depending on the synthetic assay substrate used (Bussink et al., 2009). Similarly, it has been determined that the A442V SNP decreases the enzymatic activity of expressed chitotriosidase(Lee et al., 2007), while the A442G SNP may be associated with increased risk of atopic disorders in childhood (Kim et al., 2013).

**Fig. 5:**
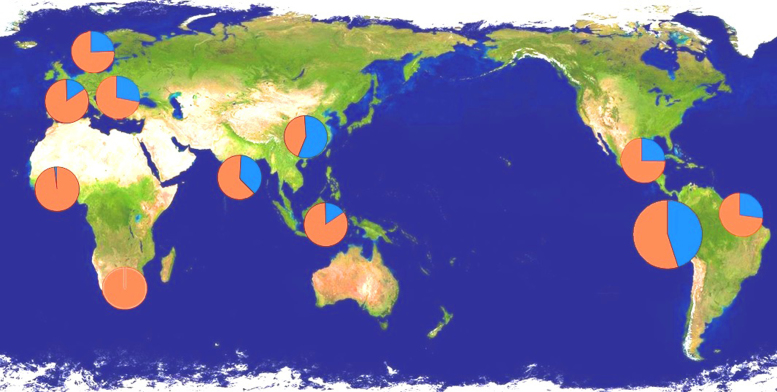
*CHIT1* duplication distribution in human populations (in blue the 24-bp duplication, in red the Wt). Allele frequencies represented in the map are (from left to right) for the following countries: Burkina Faso (Wt freq.: 98%) (Malaguarnera et al., 2003), South Africa (100%) (Arndt et al., 2013), Spain (Basques) (88%) (Piras et al., 2007a, Piras et al., 2007b), The Netherlands (77%) (Boot et al., 1998), Sicily (73%) (Piras et al., 2007a, Piras et al., 2007b), India (60%) (Choi et al., 2001), Papua New Guinea (88%) (Hise et al., 2003), Chinese Han, Taiwan (42%) (Chien, Chen, & Hwu, 2005), Mexico (76%) (Juárez-Rendón, Lara-Aguilar, & García-Ortiz, 2013), Brazil (74%) (Adelino et al., 2013) and Amerindians of Peru (52%). (For interpretation of the references to color in this figure legend, the reader is referred to the web version of this article.)

No significant deviation from expected genotypic frequencies under HWE were found for any of the three studied polymorphisms, as also found in several other human populations (Piras et al., 2007a, Piras et al., 2007b). This suggests that there is no strong selective pressure against the homozygous mutated phenotypes. In previous work, we proposed that human expansion to temperate regions, and the gradual improvement of hygienic conditions, may have reduced exposure to chitin from parasites and diet enabling the conservation of dysfunctional mutations in the *CHIT1* gene (Cozzarini et al., 2009, Paoletti et al., 2007). However, the current data from Peruvian Amerindians with high enteroparasite prevalence do not support this hypothesis. This finding is in line with our previous inability to find a correlation between *CHIT1* genotype and susceptibility to hookworm infection (Hall et al., 2007) (Fig. 5).

Considering that the first inhabitants of the New World descended from eastern Asian populations between 6 and 30 thousand years ago, it is plausible to assume that the high frequencies of the mutation (>40%) result from a founder effect in Amerindians. Both linguistic and genetic data suggest that the populations considered in our study are reciprocally isolated: first, *Awajún*, *Ashaninka*, *Shipibo* and *Quechua* belong to four different linguistic macro-families, being separated at higher levels of classification (Cavalli-Sforza, Piazza, Menozzi, & Mountain, 1988); second, population genetic information based on different genetic markers in South-Amerindians confirm significant genetic divergence between the ethno-linguistic groups considered in this study (Bisso-Machado, Bortolini, & Salzano, 2012). Thus, our relatively small sample of ‘Peruvian Amerindians’ is more broadly representative of ‘South American Amerindians’.

Chitotriosidase has been suggested to have a role in resistance to malaria, as the H-allele is almost absent in highly malarious areas of Africa. An anti-malarial role of the 24-bp duplication has been hypothesized for European and Mediterranean populations living in regions endemic for malaria until recent times (Piras et al., 2007a, Piras et al., 2007b). The interaction mechanism proposed by Di Luca and colleagues (Di Luca et al., 2007), concerns the fact that *Plasmodium*'s chitinase is required for mosquito's peritrophic membrane digestion. Human CHIT1, which is increased in blood during acute infection (Barone, Simporé, Malaguarnera, Pignatelli, & Musumeci, 2003), possibly contributes to this process (Di Luca et al., 2007). Therefore, the catalytically inactive form of human CHIT1 has been suggested to reduce malaria transmission at subsequent bites, thus high frequencies of the duplication may confer some level of protection against malaria at the community level. Indeed, a positive association between H-allele frequency and altimetric distribution of malaria was found in Sardinian and Sicilian cohorts (Piras et al., 2007b).

However, our previous study in Papua New Guinea failed to find a correlation between *CHIT1* genotype and malarial status in a *P. falciparum* mesoendemic region (Hall et al., 2007). As noted by previous authors, the inactivated, transmission-reducing CHIT1 may not be required in Benin and Burkina Faso populations, which display high frequencies for genetic conditions conferring natural resistance against malarial pathogenicity, such as thalassemia and G6PDH deficiency (Malaguarnera et al., 2003), as well as Duffy factor negativity (Gething et al., 2012), that may encourage selection for ‘anti-parasitic’ (enzymatically active) CHIT1 rather than ‘anti-malarial’ (enzymatically inactive) CHIT1. However, the duplication was present in a Papua New Guinea population with high malaria and a high frequency of deleterious malaria resistance genes (Hall et al., 2007).

Historical and epidemiological data suggest *Plasmodium* species spread in the New World after the European conquest (De Castro & Singer, 2005), and malaria became mesoendemic in the Peruvian Amazon lowlands at the beginning of the 17th century. Moreover, malaria vector distribution in Peru is strictly limited by altitude and *Quechua* populations living in the highlands (>2500 m above sea level) thus are not exposed to this pathogen. In our study, an effect of altitude could not be confirmed as the duplication resulted conserved in *Quechua-Cusco* and *Quechua-Lamas*, similarly to Amazonians. However, known rates of malarial infection in Peru are low in comparison to West African population (Malaguarnera et al., 2003) and other South American countries, even amongst non-indigenous populations (Gething et al., 2012), therefore it is not excluded that the 24-bp duplication, conserved as founder effect amongst Amerindian populations, may secondarily control malaria transmission, and high frequencies of the mutant allele may contribute in controlling the spread of malaria in the Amazon.

Finally, it must be considered that a significant prevalence of the two SNPs considered, together with other variants causing chitotriosidase deficiency (SNP G354R; deletion: E/I-10 delGAgt) (Grace, Balwani, Nazarenko, Prakash-Cheng, & Desnick, 2007) has been described in Black South Africans (Arndt et al., 2013), where the 24-bp duplication is absent. Thus, these authors inferred that the loss of chitinolytic function has arisen in modern humans by different genetic events.

## 5 Conclusions

Many scientists investigating bio-anthropological issues in isolated ethnic populations have stressed the need for studying specific epidemiological and immunological features to adopt appropriate health-care interventions (i.e. Hurtado et al., 2005, Hurtado, Frey, Hurtado, Hill, & Baker, 2008). Our evidence shows that in Amazonian Amerindians, who have been living relatively isolated until only a few decades ago, *CHIT1* genotypes are similar to those found in Asian populations, in marked contrast to African and European populations. The high frequency of the dysfunctional *CHIT1* genotypes is consistent with a founder effect and does not corroborate previous suggestions of a persisting anti-parasitic function of this gene. However, demographic and environmental conditions in the Amazon are rapidly changing, and new epidemic burdens are arising (e.g. filariasis in Brazilian Amazon or zoonotic infections from the introduction of cattle in indigenous areas), therefore new immunoparasitological challenges may impact on Amazonian populations with high prevalence of chitinase deficiency, in the near future.

Chitotriosidase expression and activity have been suggested as efficient markers of immune-mediated disorders as Gaucher's, Atherosclerosis or Alzheimer's (Malaguarnera et al., 2003, Grace et al., 2007), but the high prevalence of chitotriosidase deficiency in our South Amerindian sample precludes this diagnostic application in South American subjects with marked ethnic ancestry.

## References

[bib0005] Adelino T.E.R., Martins G.G., Gomes A.A.A., Torres A.A., Silva D.A.S., Xavier V.D.O. (2013). Biochemical and molecular chitotriosidase profiles in patients with Gaucher disease Type 1 in Minas Gerais, Brazil: New mutation in CHIT1 gene. JIMD Reports.

[bib0010] Araujo A., Reinhard K.J., Ferreira L.F., Gardner S.L. (2008). Parasites as probes for prehistoric human migrations?. Trends in Parasitology.

[bib0015] Arndt S., Hobbs A., Sinclaire I., Lane A.B. (2013). Chitotriosidase deficiency: A mutation update in an African population. JIMD Reports.

[bib0020] Arroyo-Begovich A., Carabez-Trejo A., de la Torre M. (1980). Staining of cysts of *Entamoeba invadens*, *Entamoeba histolytica* and *Entamoeba coli* with wheat germ agglutinin labelled with colloidal gold. Archivos de Investigación Médica.

[bib0025] Barone R., Simporé J., Malaguarnera L., Pignatelli S., Musumeci S. (2003). Plasma chitotriosidase activity in acute *Plasmodium falciparum* malaria. Clinica Chimica Acta: International Journal of Clinical Chemistry.

[bib0030] Bierbaum S., Superti-Furga A., Heinzmann A. (2006). Genetic polymorphisms of chitotriosidase in Caucasian children with bronchial asthma. International Journal of Immunogenetics.

[bib0035] Bisso-Machado R., Bortolini M.C., Salzano F.M. (2012). Uniparental genetic markers in South Amerindians. Genetics and Molecular Biology.

[bib0040] Boot R.G., Bussink A.P., Verhoek M., de Boer P.A.J., Moorman A.F.M., Aerts J.M.F.G. (2005). Marked differences in tissue-specific expression of chitinases in mouse and man. The Journal of Histochemistry and Cytochemistry.

[bib0045] Boot R.G., Renkema G.H., Verhoek M., Strijland a., Bliek J., de Meulemeester T.M. (1998). The human chitotriosidase gene. Nature of inherited enzyme deficiency. The Journal of Biological Chemistry.

[bib0050] Bourgain C. (2005). Comparing strategies for association mapping in samples with related individuals. BioMed Central Genetics.

[bib0055] Boussac M., Garin J. (2000). Calcium-dependent secretion in human neutrophils: A proteomic approach. Electrophoresis.

[bib0060] Brydon L.J., Gooday G.W., Chappell L.H., King T.P. (1987). Chitin in egg shells of *Onchocerca gibsoni* and *Onchocerca volvulus*. Molecular and Biochemical Parasitology.

[bib0065] Bussink A.P., Verhoek M., Vreede J., Ghauharali-van der Vlugt K., Donker-Koopman W.E., Sprenger R.R. (2009). Common G102S polymorphism in chitotriosidase differentially affects activity towards 4-methylumbelliferyl substrates. The FEBS Journal.

[bib0070] Cavalli-Sforza L.L., Piazza A., Menozzi P., Mountain J. (1988). Reconstruction of human evolution: Bringing together genetic, archaeological, and linguistic data. Proceedings of the National Academy of Sciences of the United States of America.

[bib0075] Chien Y.-H., Chen J.-H., Hwu W.-L. (2005). Plasma chitotriosidase activity and malaria. Clinica Chimica Acta: International Journal of Clinical Chemistry.

[bib0080] Choi E.H., Zimmerman P.A., Foster C.B., Zhu S., Kumaraswami V., Nutman T.B. (2001). Genetic polymorphisms in molecules of innate immunity and susceptibility to infection with *Wuchereria bancrofti* in South India. Genes and Immunity.

[bib0085] Cozzarini E., Bellin M., Norberto L., Polese L., Musumeci S., Lanfranchi G. (2009). CHIT1 and AMCase expression in human gastric mucosa: Correlation with inflammation and Helicobacter pylori infection. European Journal of Gastroenterology & Hepatology.

[bib0090] De Castro M.C., Singer B.H. (2005). Was malaria present in the Amazon before the European conquest?. Available evidence and future research agenda. Journal of Archaeological Science.

[bib0095] Di Luca M., Romi R., Severini F., Toma L., Musumeci M., Fausto A.M. (2007). High levels of human chitotriosidase hinder the formation of peritrophic membrane in anopheline vectors. Parasitology Research.

[bib0100] Gething P.W., Elyazar I.R.F., Moyes C.L., Smith D.L., Battle K.E., Guerra C.A. (2012). A long neglected world malaria map: *Plasmodium vivax* endemicity in 2010. PLoS Neglected Tropical Diseases.

[bib0105] Gianfrancesco F., Musumeci S. (2004). The evolutionary conservation of the human chitotriosidase gene in rodents and primates. Cytogenetic and Genome Research.

[bib0110] Gordon-Thomson C., Kumari a., Tomkins L., Holford P., Djordjevic J.T., Wright L.C. (2009). Chitotriosidase and gene therapy for fungal infections. Cellular and Molecular Life Sciences.

[bib0115] Grace M.E., Balwani M., Nazarenko I., Prakash-Cheng A., Desnick R.J. (2007). Type 1 Gaucher disease: Null and hypomorphic novel chitotriosidase mutations-implications for diagnosis and therapeutic monitoring. Human Mutation.

[bib0120] Hall A.J., Morroll S., Tighe P., Götz F., Falcone F.H. (2008). Human chitotriosidase is expressed in the eye and lacrimal gland and has an antimicrobial spectrum different from lysozyme. Microbes and Infection.

[bib0125] Hall A.J., Quinnell R.J., Raiko A., Lagog M., Siba P., Morroll S. (2007). Chitotriosidase deficiency is not associated with human hookworm infection in a Papua New Guinean population. Infection, Genetics and Evolution.

[bib0130] Harrington B.J. (2008). Microscopy of 4 pathogenic enteric protozoan parasites: A review. LabMedicine.

[bib0135] Hise A.G., Hazlett F.E., Bockarie M.J., Zimmerman P.A., Tisch D.J., Kazura J.W. (2003). Polymorphisms of innate immunity genes and susceptibility to lymphatic filariasis. Genes and Immunity.

[bib0140] Hurtado A.M., Frey M.A., Hurtado I., Hill K., Baker J., Elton S., O’Higgins P. (2008). Medicine and evolution: Current applications, future prospects.

[bib0145] Hurtado A.M., Lambourne C.A., James P., Hill K., Cheman K., Baca K. (2005). Human rights, biomedical science and infectious diseases among South American Indigenous Groups. Annual Review of Anthropology.

[bib0150] Instituto Nacional de Salud (2000).

[bib0155] Instituto Nacional de Salud (2003).

[bib0160] Irún P., Alfonso P., Aznarez S., Giraldo P., Pocovi M. (2013). Chitotriosidase variants in patients with Gaucher disease. Implications for diagnosis and therapeutic monitoring. Clinical Biochemistry.

[bib0165] Jongedijk E., Tigelaar H., Roekel J.S.C., Bres-Vloemans S.A., Dekker I., Elzen P.J.M. (1995). Synergistic activity of chitinases and β-1,3-glucanases enhances fungal resistance in transgenic tomato plants. Euphytica.

[bib0170] Juárez-Rendón K.J., Lara-Aguilar R.A., García-Ortiz J.E. (2013). 24-bp Duplication on CHIT1 gene in Mexican population. Revista Médica Del Instituto Mexicano Del Seguro Social.

[bib0175] Kasprzewska A. (2003). Plant chitinases-regulation and function. Cellular & Molecular Biology Letters.

[bib0180] Kim K.W., Park J., Lee J.H., Lee H.S., Lee J., Lee K.-H. (2013). Association of genetic variation in chitotriosidase with atopy in Korean children. Annals of Allergy, Asthma & Immunology.

[bib0185] Lanuza M.D., Carbajal J.A., Borrás R. (1996). Identification of surface coat carbohydrates in *Blastocystis hominis* by lectin probes. International Journal for Parasitology.

[bib0190] Lee P., Waalen J., Crain K., Smargon A., Beutler E. (2007). Human chitotriosidase polymorphisms G354R and A442V associated with reduced enzyme activity. Blood Cells, Molecules & Diseases.

[bib0195] Malaguarnera L., Simporè J., Prodi D.A., Angius A., Sassu A., Persico I. (2003). A 24-bp duplication in exon 10 of human chitotriosidase gene from the sub-Saharan to the Mediterranean area: Role of parasitic diseases and environmental conditions. Genes and Immunity.

[bib0200] Marquis G., Montplaisir S., Garzon S., Strykowski H., Auger P. (1982). Fungitoxicity of muramidase. Ultrastructural damage to *Candida albicans*. Laboratory Investigation.

[bib0205] MINSA/OGE (2002).

[bib0210] MINSA/OGE (2003).

[bib0215] Muzzarelli R.A.A., Gupta N.S. (2011).

[bib0220] Muzzarelli R., Boudrant J., Meyer D., Manno N., Demarchis M., Paoletti M. (2012). Current views on fungal chitin/chitosan, human chitinases, food preservation, glucans, pectins and inulin: A tribute to Henri Braconnot, precursor of the carbohydrate polymers science, on the chitin bicentennial. Carbohydrate Polymers.

[bib0224] Musumeci S. & Paoletti M.G. (eds.) Binomium Chitin-Chitinase - recent issues. Nova Science Publishers. 2009.

[bib0225] Paoletti M.G., Norberto L., Damini R., Musumeci S. (2007). Human gastric juice contains chitinase that can degrade chitin. Annals of Nutrition & Metabolism.

[bib0230] Piras I., Falchi A., Melis A., Ghiani M.E., Calò C.M., Varesi L. (2007). 24 bp duplication of CHIT1 gene is not correlated with coronary artery disease in Corsica Island (France). Experimental and Molecular Pathology.

[bib0235] Piras I., Melis A., Ghiani M.E., Falchi A., Luiselli D., Moral P. (2007). Human CHIT1 gene distribution: New data from Mediterranean and European populations. Journal of Human Genetics.

[bib0240] Ritchie L.S. (1948). An ether sedimentation technique for routine stool examinations. Bulletin of the U.S. Army Medical Department.

[bib0245] Rodrigues M.R., Sá Miranda M.C., Amaral O. (2004). Allelic frequency determination of the 24-bp chitotriosidase duplication in the Portuguese population by real-time PCR. Blood Cells, Molecules & Diseases.

[bib0250] Seibold M.a., Donnelly S., Solon M., Innes A., Woodruff P.G., Boot R.G. (2008). Chitotriosidase is the primary active chitinase in the human lung and is modulated by genotype and smoking habit. The Journal of Allergy and Clinical Immunology.

[bib0255] Sheather A. (1923). The detection of intestinal protozoa and mange parasites by a flotation technique. Journal of Comparative Pathology.

[bib0260] Sianto L., Chame M., Silva C.S.P., Gonçalves M.L.C., Reinhard K., Fugassa M. (2009). Animal helminths in human archaeological remains: A review of zoonoses in the past. Revista Do Instituto de Medicina Tropical de São Paulo.

[bib0265] Van Eijk M., van Roomen C.P.a.a., Renkema G.H., Bussink A.P., Andrews L., Blommaart E.F.C. (2005). Characterization of human phagocyte-derived chitotriosidase, a component of innate immunity. International Immunology.

[bib0270] Vandevenne M., Campisi V., Freichels A., Gillard C., Gaspard G., Frère J.-M. (2011). Comparative functional analysis of the human macrophage chitotriosidase. Protein Science.

[bib0275] Verwer P.E.B., Ten Kate M.T., Falcone F.H., Morroll S., Verbrugh H.A., Bakker-Woudenberg I.A.J.M. (2013). Evidence supporting a role for mammalian chitinases in efficacy of caspofungin against experimental aspergillosis in immunocompromised rats. PloS One.

[bib0280] Wimmer M., Schmid B., Tag C., Hofer H.W. (1998). *Ascaris suum*: Protein phosphotyrosine phosphatases in oocytes and developing stages. Experimental Parasitology.

[bib0285] Woo K.H., Lee B.H., Heo S.H., Kim J.-M., Kim G.-H., Kim Y.-M. (2014). Allele frequency of a 24-bp duplication in exon 10 of the CHIT1 gene in the general Korean population and in Korean patients with Gaucher disease. Journal of Human Genetics.

[bib0290] Zakrzewski A.-C., Weigert A., Helm C., Adamski M., Adamska M., Bleidorn C. (2014). Early divergence, broad distribution, and high diversity of animal chitin synthases. Genome Biology and Evolution.

[bib0295] Zhang Y., Foster J.M., Nelson L.S., Ma D., Carlow C.K.S. (2005). The chitin synthase genes chs-1 and chs-2 are essential for *C. elegans* development and responsible for chitin deposition in the eggshell and pharynx, respectively. Developmental Biology.

